# Endovascular Treatment of Small Ruptured Intracranial Aneurysms (<5 mm): Long-term Clinical and Angiographic Outcomes and Related Predictors

**DOI:** 10.1007/s00062-019-00835-8

**Published:** 2019-11-06

**Authors:** Fei Peng, Xin Feng, Xin Tong, Baorui Zhang, Luyao Wang, Erkang Guo, Peng Qi, Jun Lu, Zhongxue Wu, Daming Wang, Aihua Liu

**Affiliations:** 1 Beijing Neurosurgical Institute, Capital Medical University, 100070 Beijing, China; 2 Department of Neurosurgery, Beijing Hospital, National Center of Gerontology, No. 1 DaHua Road, Dong Dan, 100730 Beijing, China; 3 Department of Interventional Neuroradiology, Beijing Tiantan Hospital, Capital Medical University, 100070 Beijing, China; 4 China National Clinical Research Center for Neurological Diseases, No. 119, South 4th Ring West Road, Fengtai District, 100070 Beijing, China; 5 Graduate School of Peking Union Medical College, No. 9 Dongdansantiao, Dongcheng District, 100730 Beijing, China

**Keywords:** Intracranial aneurysms, Treatment, Risk factors, Angiography, Retreatment

## Abstract

**Purpose:**

To investigate the long-term clinical and angiographic outcomes and their related predictors in endovascular treatment (EVT) of small (<5 mm) ruptured intracranial aneurysms (SRA).

**Methods:**

The study retrospectively reviewed patients with SRAs who underwent EVT between September 2011 and December 2016 in two Chinese stroke centers. Medical charts and telephone call follow-up were used to identify the overall unfavorable clinical outcomes (OUCO, modified Rankin score ≤2) and any recanalization or retreatment. The independent predictors of OUCO and recanalization were studied using univariate and multivariate analyses. Multivariate Cox proportional hazards models were used to identify the predictors of retreatment.

**Results:**

In this study 272 SRAs were included with a median follow-up period of 5.0 years (interquartile range 3.5–6.5 years) and 231 patients with over 1171 aneurysm-years were contacted. Among these, OUCO, recanalization, and retreatment occurred in 20 (7.4%), 24 (12.8%), and 11 (7.1%) patients, respectively. Aneurysms accompanied by parent vessel stenosis (AAPVS), high Hunt-Hess grade, high Fisher grade, and intraoperative thrombogenesis in the parent artery (ITPA) were the independent predictors of OUCO. A wide neck was found to be a predictor of recanalization. The 11 retreatments included 1 case of surgical clipping, 6 cases of coiling, and 4 cases of stent-assisted coiling. A wide neck and AAPVS were the related predictors.

**Conclusion:**

The present study demonstrated relatively favorable clinical and angiographic outcomes in EVT of SRAs in long-term follow-up of up to 5 years. THE AAPVS, as a morphological indicator of the parent artery for both OUCO and retreatment, needs further validation.

## Introduction

As reported by experienced neurosurgeons and interventionalists, most ruptured aneurysms are small in size [1, 2]. Subarachnoid hemorrhage (SAH) is more extensive in these aneurysms than in the larger aneurysms [3]. Small (<5 mm) ruptured intracranial aneurysms (SRAs) have recently drawn attention, as they accounted for nearly 50% of SAH cases [4, 5]. A 25-year single center study reported that the proportion of SRAs increased from 29% in the first 5‑year period to 50% in the most recent interval [4].

Endovascular treatment (EVT) has been elucidated as a beneficial treatment modality for ruptured intracranial aneurysms (RIAs): however, due to technical challenges including the stability of the microcatheter position, the coil conformability, and the reliability of coil detachment, complication rates may increase in EVT of small RIAs (<5 mm) [6, 7]. Intraprocedural rupture (IPR) rates in very small (≤3 mm) RIAs ranged from 7.7% to 11.7%, and these rates were 2–5 times higher than those in the larger aneurysms [8, 9]; however, previous studies regarding the small RIAs were mainly focused on the very small (≤3 mm) aneurysms associated with short-term follow-up periods and a limited sample size [10–12]. Long-term clinical and angiographic outcomes in EVT of SRAs (<5 mm) and discrepancies between the ≤3 mm and 3–5 mm aneurysms still remain poorly defined [10].

In a preliminary study the clinical and the angiographic outcomes were found to be acceptable in the EVT of small (<5 mm) unruptured intracranial aneurysms (UIAs) [13]; however, the clinical and angiographic outcomes may vary from the UIAs to the RIAs considering the inherent heterogeneity and different therapeutic regimens. In the present study, a retrospective analysis of consecutive patients with SRAs who received EVT in two Chinese stroke centers was conducted to clarify the long-term clinical and angiographic outcomes in EVT of SRAs and related factors.

## Methods

### Study Population and Data Collection

Patients with saccular SRAs who underwent EVT between February 2011 and December 2016 in two Chinese stroke centers (Beijing Tiantan Hospital and Beijing Hospital) were retrospectively enrolled in the long-term follow-up multicenter databases. The operators enrolled in this study were skilled and had more than 10 years experience in EVT, with every operator performing more than 100 EVTs for intracranial aneurysms each year. The present study was authorized by the Institutional Review Board of Beijing Tiantan Hospital. Patients with (1) multiple aneurysms where the aneurysm responsible for SAH could not be identified, (2) incomplete data of medical charts or angiographic information, (3) fusiform, dissecting, traumatic, and blood blister-like aneurysms or aneurysms associated with arteriovenous fistulas, arteriovenous malformations, or Moyamoya disease were excluded.

Patients data were retrospectively collected from medical charts and angiographic records. Patient characteristics included age, sex, history of coronary artery disease, dyslipidemia, hypertension and diabetes mellitus, alcohol consumption habit, smoking status (never smoked, current smokers, and former smokers), prehospital delay after SAH ictus, preprocedure delay after SAH, previous aneurysmal SAH and the treatment modality (coiling, stent-assisted or balloon-assisted coiling). Aneurysm characteristics included size (also classified into 3 mm and 3–5 mm) and location, aspect ratio (AR), small basal outpouching (SBO), aneurysms accompanied with parent vessel stenosis (AAPVS; the degree of stenosis of 50%, 50–70%, and >70% were classified as no, mild, and severe AAPVS, respectively), wide neck aneurysms (aneurysm neck ≥4 mm or AR <1.3), and aneurysm shape (regularity, lobe, bleb, and other irregularities). The locations of the aneurysms were divided into four categories: (1) distal vessels (distal branches such as A2, M2, P2, and beyond) [14], (2) vascular eloquence (VE, first segments of the cerebral arteries such as A1, M1, P1 segments, parent arteries less than 20 mm away from the internal carotid artery, important vessel branches, such as the posterior inferior cerebellar artery and the anterior choroidal artery that support the brain stem and the basal ganglia, or cerebral bifurcation arteries, such as the basilar artery and the internal carotid artery bifurcation) [15, 16], (3) communicating arteries (the anterior communicating artery and the posterior communicating artery), and (4) other locations. An SBO was defined as an aneurysmal daughter sac or bleb located near the base of the aneurysm [17].

### Endovascular Treatment

Surgery was carried out with the patients under general anesthesia and systemic heparinization, with an aim to maintain the activated clotting time at twice the baseline value. Heparinization was started when the aneurysm was partially occluded and the first or more coils were deployed [18]. Wide-necked aneurysms were coiled with either a stent or a balloon according to the operators’ preferences. Patients were administered 300 mg clopidogrel and 300 mg aspirin during the procedure of the stent-assisted coiling. In the case of an intraprocedural rupture, rapid coil deposition was often considered, heparin was reversed by protamine sulphate, and a balloon was inflated near the proximal artery if needed. Glycoprotein IIb/IIIa inhibitor (Tirofiban, Grand Pharma, Wuhan, China) was used in acute thrombotic events. In the postprocedural period, clopidogrel (75 mg/day) was continued for 6 weeks and aspirin (100 mg/day) was continued for at least 6 months.

### Clinical and Angiographic Follow-up

The perioperative complications were defined as procedure-related hemorrhagic complications including IPR and early aneurysmal rebleeding, and procedure-related ischemic events, such as early postprocedural infarctions (EPPI) and intraoperative thrombogenesis in the parent artery (ITPA).

The follow-up information was obtained by telephone survey to make a detailed assessment of the record of modified Rankin scale (mRS) scores, the clinical events during the follow-up period, such as any late ischemic events (defined as postprocedural infarctions or thromboembolic events during the follow-up period), late hemorrhagic strokes (defined as postprocedural hemorrhages during the follow-up period), any retreatments for the treated aneurysm, and the survival condition (death or living) of the patients. The causes of death were classified into three categories: cerebrovascular-related, cancer-related, and all-cause mortality. The patients were evaluated with the mRS score at discharge and at 6‑month, 1‑year, 2‑year, and 5‑year follow-up. Good and poor clinical outcomes were defined as mRS scores of 0–2 and 3–6, respectively. The overall unfavorable clinical outcomes (OUCO) were defined as poor outcomes at the latest available follow-up. The mRS scores of 3–5 or at least 1‑point increment during hospitalization or during the follow-up were considered as morbidity.

Computed tomography angiography (CTA), magnetic resonance angiography (MRA), and/or digital subtraction angiography (DSA) were performed for the patients at 6 months, 1 year, 2 years, and 5 years after discharge. The occlusion rates were estimated by the Raymond scale (RS) (RS1 was defined as complete obliteration, RS2 as residual neck and RS3 as residual aneurysm) immediately after the EVT and at the follow-up [19]. The RS1 and RS2 grades were considered as satisfactory outcomes. The follow-up angiographic results were classified into four categories when compared with the immediate degree of embolization: occluded, improved, stable and recanalized, manifested as no contrast material, decreased contrast material, unchanged contrast material and increased contrast material filling into the aneurysm sac, respectively. Recanalization was defined as decreasing percentage of occlusion in the follow-up angiography. Retreatment was defined as retreatment by EVT in the treated aneurysm.

### Statistical Analysis

The time range between discharge and the first retreatment was used for the survival analyses. Log-rank testing and multivariate Cox proportional hazards models were used for survival analyses of the retreatment. Univariate analysis and logistic regression were used to identify the independent predictors of OUCO and recanalization. Continuous variables were analyzed using the Mann-Whitney U-test and the categorical variables were analyzed using the Pearson χ^2^-test. Any variable with a *P*-value <0.20 in the univariate analysis was entered into the multivariate logistic regression model. A *P*-value <0.05 was considered statistically significant. The SPSS version 23.0 (SPSS, Chicago, Ill, USA) was used for all statistical analyses.

## Results

A total of 271 patients harboring 272 SRAs who underwent EVT were included in the databases. Ages of the patients with SRAs ranged from 14 to 78 years with a median age of 53.5 ± 0.69 years and 54.6% of the patients were female. The baseline characteristics of the patients and aneurysms are listed in Table 1.

**Table 1:** Baseline characteristics of 271 patients and 272 ruptured aneurysms

Characteristics	
Mean ± SD age (years)	53.4 ± 11.6
Female gender	148 (54.4%)
Hunt and Hess grade (>2)	40 (14.7%)
Fisher grade (>2)	58 (21.3%)
*Shape of aneurysm*	
Regular	128 (47.1%)
Lobular	11 (4.0%)
Daughter sac	50 (18.4%)
Other irregularity	83 (30.5%)
VE	58 (21.3%)
SBO	39 (14.3%)
AAPVS	
None	235 (86.4%)
Mild	30 (11.0%)
Severe	7 (2.6%)

*VE* vascular eloquence (parent artery was <20 mm from the internal carotid artery or the first segment of cerebral arteries, e.g., A1, M1, P1 segments), *SBO* small basal outpouching, *AAPVS* aneurysms accompanied with the parent vessel stenosis

### Clinical Outcomes at Discharge and During the Follow-up

At discharge, 230 out of 271 patients (84.9%) had good outcomes (mRS ≤ 2). During the follow-up period 231 (85.2%) patients could be contacted. The total follow-up time was 1171 aneurysm-years, and the mean follow-up time was 5.0 years (interquartile range 3.5–6.5 years). Clinical outcomes were good (mRS ≤ 2) in 215 (93.1%) out of 231 patients at the 6‑month follow-up, in 214 (92.6%) patients out of 231 at the 1‑year follow-up, in 215 (93.5%) patients out of 230 at the 2‑year follow-up, and in 117 (90.0%) patients out of 130 at the 5‑year follow-up. An OUCO was observed in 20 cases, and the morbidity and the mortality rates were 7.4% and 4.4%, respectively. Of the patients four had late postprocedural infarctions. The infarctions occurred in the first month, after 10 months, the first year, and the second year, respectively after discharge in these patients. Among them, one patient lived with permanent morbidity (mRS = 5) and the other three lived without morbidity (mRS ≤ 2). Of the patients three patients had postprocedural hemorrhages, two of them occurred in the second year after discharge, one of them was due to rerupture of the aneurysm and the other was due to intraparenchymal hemorrhage. Both patients lived with permanent morbidity (mRS = 4). The third instance of hemorrhage occurred in the fifth year after discharge and the patient died of cerebrovascular related reason. Of the 271 patients 12 (4.4%) died (mRS = 6), 8 deaths occurred in the perioperative period of which 4 were cerebrovascular related and the other 4 were all-cause mortality. Of the deaths 4 (1.7%) occurred in the 1171 aneurysm-years, and the annual mortality rate was 0.34%. Out of these four deaths two were all-cause mortality that occurred in the second year and in the fourth year, respectively. The remaining two deaths occurred in the fifth year and they were cerebrovascular-related and cancer related, respectively.

After the multivariate analyses, the predictors of OUCO were AAPVS (odds ratio, OR 13.258, 95% confidence interval, CI: 2.089–84.132, *P* = 0.006), high Hunt-Hess grade (OR 3.811, 95% CI 1.108–13.111, *P* = 0.034), high Fisher grade (OR 3.520, 95% CI 1.042–11.887, *P* = 0.043), and ITPA (OR 17.239, 95% CI 1.015–292.930, *P* = 0.049) (Table 3).

**Table 2:** Potential risk factors related to recanalization

Characteristics	Recanalization	Univariate	Multivariate	
			OR (95%CI)	*P* value
Female	14 (9.0%)	0.359	–	–
Age (≥50 years)	14 (9.0%)	0.647	–	–
Acute stage	7.1%	1.000	–	–
Preprocedural delay after SAH	–	0.546	–	–
≤3 days	10 (6.4%)	–	–	–
3–14 days	7 (4.5%)	–	–	–
15–28 days	2 (1.3%)	–	–	–
>28 days	3 (1.9%)	–	–	–
History of SAH	1 (0.6%)	0.202	–	–
Hunt-Hess	–	0.502	–	–
1–2	18 (11.5%)	–	–	–
>2	4 (2.6%)	–	–	–
Fisher	–	1.000	–	–
1–2	19 (12.2%)	–	–	–
>2	3 (1.9%)	–	–	–
Hypertension	13 (8.3%)	1.000	–	–
Hyperlipidemia	7 (4.5%)	0.801	–	–
Diabetes mellitus	2 (1.3%)	1.000	–	–
Coronary artery disease	2 (1.3%)	0.655	–	–
Drinking status	3 (1.9%)	0.194	0.340 (0.044–2.640)	0.302
Smoking status	–	0.057	3.481 (0.501–24.193)	0.207
Never smoking	18 (11.5%)	–	–	–
Current smoking	2 (1.3%)	–	–	–
Former smoking	2 (1.3%)	–	–	–
Aneurysm location	–	0.859	–	–
Distal vessels	1 (0.7%)	–	–	–
Communicating arteries	4 (2.6%)	–	–	–
VE	3 (2.0%)	–	–	–
Other arteries	11 (7.3%)	–	–	–
Shape of aneurysm	–	0.635	–	–
Regular	11 (7.1%)	–	–	–
Lobular	6 (3.8%)	–	–	–
Daughter sac	0 (0.0%)	–	–	–
Others	5 (14.1%)	–	–	–
AAPVS	–	0.109	0.674 (0.119–3.829)	0.656
None	18 (11.5%)	–	–	–
Mild	2 (1.3%)	–	–	–
Severe	2 (1.3%)	–	–	–
VE	5 (3.2%)	1.000	–	–
SBO	2 (1.3%)	0.741	–	–
Multiplicity	3 (1.9%)	1.000	–	–
Aneurysm size	–	0.220	–	–
≤3 mm	4 (2.6%)	–	–	–
3–5 mm	18 (11.5%)	–	–	–
Treatment mortality	–	0.133	0.154 (0.023–1.055)	0.057
Wide neck	–	0.011	0.318 (0.117–0.864)	0.025
Yes	5 (3.2%)	–	–	–
No	17 (10.9%)	–	–	–
Coiling	15 (9.6%)	–	–	–
Stent-assisted coiling	4 (2.6%)	–	–	–
Balloon-assisted coiling	3 (1.9%)	–	–	–
RS score	–	0.127	2.261 (0.401–12.728)	0.355
RS1	17 (10.9%)	–	–	–
RS2	2 (1.3%)	–	–	–
RS3	3 (1.9%)	–	–	–

*OR* Odds ratio, *SAH* subarachnoid hemorrhage, *VE* vascular eloquence, *AAPVS* aneurysms accompanied with parent vessel stenosis, *SBO* small basal outpouching, *RS* Raymond scale

### Immediate and Long-term Angiographic Outcomes

Among the 272 SRAs immediate complete obliteration (RS1), residual neck (RS2), and residual aneurysm (RS3) were observed in 205 patients (75.4%), 54 patients (19.9%), and 13 patients (4.7%), respectively. The initial satisfactory occlusion was achieved in 259 patients (95.2%). Among the 263 patients who survived at discharge, 156 patients (59.3%) underwent angiographic follow-up at least once, with the follow-up period ranging from 1 month to 78 months (median 7.0 months). Follow-up angiograms showed that 102 aneurysms (65.4%) were occluded, 24 aneurysms (15.4%) were improved, 12 aneurysms (7.7%) were stable, and 22 aneurysms (14.1%) were recanalized. The annual rate of recanalization was 1.9% (22 in 1171). Among the 22 recanalizations, 16 (72.7%) occurred in the first year, 3 (13.7%) occurred in the second year, 2 (9.1%) occurred in the third year, and 1 (4.5%) occurred in the fifth year. Out of the 22 recanalizations 11 received retreatment (annual rate 0.9%, 11 in 1171). The retreatment included one case of surgical clipping, six cases of coiling, and four cases of stent-assisted coiling and two patients (18.2%, 2 in 11) lived with permanent morbidity after the retreatment. After the multivariate analyses, wide neck was found to be the predictor of recanalization (OR 0.318, 95% CI 0.117–0.864, *P* = 0.025) (Table 2).

**Fig. 1:**
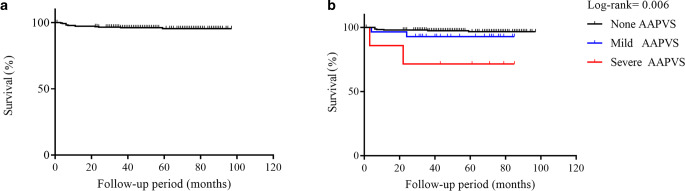
**a** Kaplan-Meier curve of the survival function for retreatment. Mean survival: 49.4 ± 31.2 months. **b** Kaplan-Meier curve of the survival function comparing AAPVS none, mild, severe, which demonstrate the relationship between AAPVS and retreatment (*P* = 0.006)

The Kaplan-Meier curve of the survival function in retreatment is displayed in Fig. 1. Survival analysis demonstrated that wide neck (log-rank *P* = 0.027) and AAPVS (log-rank *P* = 0.006) significantly increased the risk of retreatment. After the multivariate Cox proportional hazards analyses, wide neck (hazard ratio, HR 0.172, 95% CI 0.035–0.831, *P* = 0.029) and AAPVS (HR 12.549, 95% CI 2.536–62.108, *P* = 0.002) remained significant.

Statistical analysis demonstrated that there were no discrepancies in OUCO (*P* = 0.420), recanalization (*P* = 0.220), and retreatment (*P* = 1.000) between the ≤3 mm and the 3–5 mm group.

**Table 3:** Potential risk factors related to OUCO

Characteristics	OUCO	Univariate	Multivariate	
			OR (95%CI)	*P* value
Female	11 (4.8%)	1.000	–	–
Age (≥50 years)	14 (6.1%)	0.475	–	–
Acute stage	11 (4.8%)	1.000	–	–
Preprocedural delay after SAH	–	0.674	–	–
≤3 days	11 (4.8%)	–	–	–
3–14 days	4 (1.7%)	–	–	–
15–28 days	3 (1.3%)	–	–	–
>28 days	2 (0.9%)	–	–	–
Previous aSAH	2 (0.9%)	1.000	–	–
Hunt-Hess	–	0.001	3.811 (1.108–13.111)	0.034
1–2	11 (4.8%)	–	–	–
>2	9 (3.9%)	–	–	–
Fisher	–	<0.001	3.520 (1.042–11.887)	0.043
1–2	9 (3.9%)	–	–	–
>2	11 (4.8%)	–	–	–
Hypertension	14 (6.1%)	0.478	–	–
Hyperlipidemia	6 (2.6%)	1.000	–	–
Diabetes mellitus	1 (0.4%)	1.000	–	–
Coronary artery disease	4 (1.7%)	0.107	2.284 (0.505–10.335)	0.283
Drinking status	7 (3.0%)	0.437	–	–
Smoking status	–	0.838	–	–
Never smoking	13 (5.6%)	–	–	–
Current smoking	6 (2.6%)	–	–	–
Former smoking	1 (0.4%)	–	–	–
Aneurysm location	–	0.538	–	–
Distal vessels	1 (0.4%)	–	–	–
Communicating arteries	7 (3.1%)	–	–	–
VE	1 (0.4%)	–	–	–
Other arteries	11 (4.8%)	–	–	–
Shape of aneurysm	–	0.715	–	–
Regular	10(4.3%)	–	–	–
Lobular	4 (1.7%)	–	–	–
Daughter sac	1 (0.4%)	–	–	–
Others	5 (2.2%)	–	–	–
AAPVS	–	0.081	13.258 (2.089–84.132)	0.006
None	14 (6.1%)	–	–	–
Mild	4 (1.7%)	–	–	–
Severe	2 (0.9%)	–	–	–
VE	6 (2.6%)	0.423	–	–
SBO	3 (1.3%)	0.738	–	–
Multiplicity	2 (0.9%)	0.747	–	–
Aneurysm size	–	0.420	–	–
≤3 mm	3 (1.3%)	–	–	–
3–5 mm	17 (7.4%)	–	–	–
Wide neck	–	0.357	–	–
Yes	8 (3.5%)	–	–	–
No	12 (5.2%)	–	–	–
Treatment mortality	–	0.556	–	–
Coiling	13 (5.6%)	–	–	–
Stent-assisted coiling	7 (3.0%)	–	–	–
Balloon-assisted coiling	0 (0.0%)	–	–	–
RS score	–	0.961	–	–
RS1	15 (6.5%)	–	–	–
RS2	4 (1.7%)	–	–	–
RS3	1 (0.4%)	–	–	–
Ischemic events	–	0.019	–	–
Yes	15 (6.5%)	–	–	–
No	5 (2.2%)	–	–	–
EPPI	–	0.057	2.938 (0.668–12.928)	0.154
Yes	16 (6.9%)	–	–	–
No	4 (1.7%)	–	–	–
ITPA	–	0.020	17.239 (1.015–292.930)	0.049
Yes	18 (7.8%)	–	–	–
No	2 (0.9%)	–	–	–
IPR	–	0.109	4.100 (0.742–22.659)	0.106
Yes	3 (1.3%)	–	–	–
No	17 (7.4%)	–	–	–

*OUCO* overall unfavorable clinical outcomes, *OR* Odds ratio, *SAH* subarachnoid hemorrhage, *VE* vascular eloquence, *AAPVS* aneurysms accompanied with parent vessel stenosis, *SBO* small basal outpouching, *RS* Raymond scale, *EPPI* early post-procedural infarctions, *ITPA* intraoperative thrombogenesis in the parent artery, *IPR* intraprocedural rupture

## Discussion

According to recent reports, SRAs (<5 mm, small RIAs) constitute a large and increasing percentage of RIAs [8, 9]. Previous studies on EVT of the small RIAs were primarily focused on the very small (<3 mm) sized aneurysms; however, the initial and long-term clinical and angiographic outcomes of EVT of SRAs are still unclear. Therefore, a retrospective study was performed, which proved that EVT of SRAs was found to be efficient and safe in the long-term follow-up.

### The Overall Clinical Outcomes and Related Predictors

The long-term follow-up of International Subarachnoid Aneurysm Trial study reported that in the endovascular group 83% of the survivors after 5 years were independent (mRS ≤ 2) [20]. Yamaki et al. conducted a meta-analysis of EVT in very small (<3 mm) aneurysms and demonstrated that 79% patients lived with good neurologic outcome [21]. In the current study, among the 231 contacted patients, poor clinical outcome occurred in 20 patients, so the overall good clinical outcome rate was 91.3% (211 out of 231 patients). In total, 40 (14.8%) out of 271 patients died or were lost to follow-up (8 patients died before discharge and 32 lost to follow-up), so the actual overall rate of good clinical outcome was in the range of 77.9% (211/271) to 89.7% (243/271). Actually, as the two centers are the largest stroke centers in China, many patients were referred from local primary care centers; therefore, some patients with severe Hunt and Hess grades did not have the opportunity to be transferred to these hospitals, and may have stayed in the local hospital or died before they were admitted to the hospital. These factors may have contributed to the relative favorable clinical outcome in this study.

During 1171 aneurysm-years, a single late aneurysmal rebleeding occurred in a 73-year-old man in the second year after the coiling. The patient received surgical clipping; however, he developed permanent neurologic deficits (mRS = 4) after the procedure. The annual rate of late aneurysmal rebleeding in the present study (0.08%) is consistent with that in the previous studies, which reported a range of 0.11–0.32%; however, experience suggests that periodic angiographic follow-up is recommended.

The related predictors of OUCO were AAPVS, ITPA, high Hunt-Hess grade, and high Fisher grade. In the present study, OUCO was observed in 20 SRAs, 6 (30.0%, 6 in 20) cases were found to have AAPVS. Out of the 6 cases with AAPVS, 3 (50%) were treated with stent implantation. Previous studies have revealed that a moderate or severe stenosis of the parent artery adjacent to the aneurysm may increase the risk of intraprocedural rupture due to altered pressure by dilation of the stenosis before stent implantation [22]. Moreover, due to atherosclerosis, vessels such as the parent artery may be vulnerable to mechanical injury and may induce intraprocedural rupture, which is also related to poor clinical outcome [23, 24].

It has been reported that ITPA occurred more often in the RIAs than in the UIAs [25]. The reason for this finding maybe the restriction of antiaggregation and anticoagulation in SAH patients and the different periprocedural therapeutic regimens [18]. In the present study, ITPA occurred in three cases (1.1%), one patient died before discharge, one developed severe morbidity (mRS score = 5) at discharge and died 2 years later, and one patient survived and developed no morbidity. The reported rate of thromboembolic events in the RIAs ranged from 4.7% to 6% [26]. Permanent neurological deficit or death was observed in 3.8% of the patients with thromboembolic events [27]. The corresponding rates in the present study were 1.1% and 0.7%, respectively, which may in part be due to restricted attention for the procedural set-up which included flushing the catheters with heparinized saline, preparation of syringes, and the application of dual antiplatelet medication [19]. High Hunt-Hess grade and high Fisher grade were significantly associated with poor clinical outcomes according to several previous studies [28, 29]. The results of the present study were consistent with these results.

### Recanalization, Retreatment and Related Predictors

Recanalization poses a great risk of recurrent SAH to the patients [30]. Over the 1171 aneurysm-years, recanalization occurred in 22 cases (14.1%) and retreatment was required in 11 cases (7.1%). It has been reported that recanalization rates after EVT in the RIAs have varied significantly from 13% to 34%, with retreatment rates ranging from 4.7% to 20.8% [31]. The present results were consistent with these findings. Wide neck has been considered a predictor of recanalization and retreatment in several studies [32–34]. Coil prolapse or coil migration often occur in wide-necked aneurysms [35], which may cause incomplete occlusion. Moreover, incomplete lumens tend to be unstable and are more likely to result in coil mass compaction than the totally occluded aneurysms [33]. This enables the hemodynamic forces to impact on larger surface area of the coil mass near the aneurysmal neck [36]. Other predictors of recanalization such as rupture status, follow-up interval (>1 year), and location in the posterior circulation were not significant in the current study [37–40]. It was found that AAPVS, as a morphologic factor of the parent artery, was associated with retreatment. Clinically, to avoid vascular injury of the narrow parent artery due to the long duration of the operation surgery was meticulously carried out and as quickly as possible. This may lead to incomplete occlusion and may induce the occurrence of recanalization [34]. Among SRAs that could be studied at follow-up, the initial complete obliteration rates in SRAs with no AAPVS, mild AAPVS, and severe AAPVS were 94.8%, 88.9%, and 50.0% (*P* = 0.002), respectively. Moreover, due to the stenosis of the parent artery, blood flow velocity in the parent artery may increase, leading to further increase in the hemodynamic instability around the aneurysm and may induce recanalization. In conclusion, further investigation regarding AAPVS is necessary, as it may play an important role in the recanalization of the aneurysm and the OUCO. Other important predictors of retreatment, such as male sex, initial incomplete aneurysm occlusion, large aneurysm size, and low dome-to-neck ratio were not significant in the present study [38, 41].

### Strengths and Limitations

The authors believe that the present study is the first and the largest cohort study to investigate the OUCO, recanalization, and retreatment, and their related predictors in EVT of SRAs (<5 mm, small RIAs) with a long-term follow-up. Moreover, new predictors including SBO, vascular eloquence, and AAPVS were reported. The study also has several limitations. In acute stages very few patients would undergo high-resolution magnetic resonance imaging (HR-MRI). Thus, the differentiation between dissection and vessel stenosis only by digital subtraction angiography (DSA) may misdiagnose some dissections, even if a ruptured aneurysm concomitant with a dissection is very rare in clinical practice. When compared with the long-term follow-up in clinical outcomes, the angiographic follow-up rate and the time range were relatively lower due to the following reasons. The most seriously ill patients (mRS ≥ 4) at discharge may develop permanent morbidity and may easily be lost to follow-up. The patients with SAH often choose to go to the neurology clinic for re-examination, where CT is often recommended other than CTA or DSA. Moreover, some patients could not tolerate DSA, CTA or MRA. As a result, important angiographic follow-up data were not obtained. Another limitation of the study was the retrospective design and that the patients were enrolled only in two centers, which may have restricted the assessment accuracy of the results.

## Conclusion

The present study demonstrated relatively favorable clinical and angiographic outcomes in EVT of SRAs with a long-term follow-up of up to 5 years. The AAPVS, Hunt-Hess grade, Fisher grade, and ITPA were independent predictors of OUCO. As a morphologic indicator of the parent artery for both OUCO and retreatment, AAPVS reminds clinicians to pay attention to such lesions, and needs further validation. A wide neck was a predictor of both recanalization and retreatment but there were no significant discrepancies in OUCO, recanalization, and retreatment between the ≤3 mm and the 3–5 mm groups.

## Abbreviations

AAPVS: Aneurysms accompanied with parent vessel stenosis
AR: Aspect ratio
CTA: Computed tomography angiography
DSA: Digital subtraction angiography
EPPI: Early postprocedural infarction
EVT: Endovascular treatment
HR: Hazard ratio
IPR: Intraprocedural rupture
ITPA: Intraoperative thrombogenesis in the parent artery
MRA: Magnetic resonance angiography
OUCO: Overall unfavorable clinical outcomes
RIA: Ruptured intracranial aneurysm
RS: Raymond scale
SAH: Subarachnoid hemorrhage
SBO: Small basal outpouching
SRA: Small ruptured intracranial aneurysms
UIA: Unruptured intracranial aneurysm
VE: Vascular eloquence
